# Neonatal Hemolytic Jaundice: Causes, Diagnostic Approach, and Management

**DOI:** 10.3390/children12060666

**Published:** 2025-05-23

**Authors:** Stavroula Parastatidou, Andreas G. Tsantes, Chrysoula-Christina Emmanouil, Aikaterini Konstantinidi, Anastasia Kapetanaki, Rozeta Sokou

**Affiliations:** 1 Neonatal Intensive Care Unit, “Elena Venizelou” Maternity Hospital, 11521 Athens, Greece; 2 Laboratory of Haematology and Blood Bank Unit, “Attikon” Hospital, School of Medicine, National and Kapodistrian University of Athens, 12462 Athens, Greece; agtsantes@med.uoa.gr; 3 Microbiology Department, “Saint Savvas” Oncology Hospital, 11522 Athens, Greece; 4 Neonatal Intensive Care Unit, “Agios Panteleimon” General Hospital of Nikea, 18454 Piraeus, Greece; 5 Neonatal Department, Aretaieio Hospital, National and Kapodistrian University of Athens, 11528 Athens, Greece; rosesok@med.uoa.gr

**Keywords:** hemolytic jaundice, neonatal hyperbilirubinemia, neonatal hemolytic anemia

## Abstract

Neonatal jaundice remains a common issue in daily clinical practice that needs to be distinguished in physiologic and pathologic hyperbilirubinemia. Hemolytic causes are significant, often underrecognized contributors of pathologic hyperbilirubinemia, sometimes leading to severe complications. Both immune-mediated and non-immune hemolytic conditions are included in the differential diagnosis of neonatal hemolytic jaundice. Following the detection of hemolysis, family and pregnancy history, physical examination of the neonate, and further investigations are necessary. Established and newer laboratory methods are useful in the subsequent diagnostic approach. The optimal management of hemolytic jaundice alienates the risk of permanent neurologic damage.

## 1. Introduction

The majority of term, healthy neonates present with jaundice during the first days of life [1]. Jaundice is caused by increased serum bilirubin levels, which accumulate in tissues. Bilirubin is produced by the destruction of red blood cells (RBCs), deriving from the catabolism of hemoglobin. Bilirubin, in its indirect, unconjugated form, is transported in the circulation bound to serum albumin [2]. In the liver, bilirubin is conjugated with glucuronic acid and is subsequently excreted in the bile. The water-soluble conjugated bilirubin is eliminated from the gut through feces. A proportion of bilirubin is hydrolyzed by beta-glucuronidase to unconjugated bilirubin in the small intestine and reabsorbed (enterohepatic circulation).

Generally, in neonates, jaundice should be distinguished between physiologic and pathologic. Physiologic jaundice refers to the common, mild, and self-limiting condition presenting in otherwise healthy neonates. Physiologic jaundice typically appears after the first 24 h of life, reaches its peak at approximately 48 to 96 h, and recedes by week 2 to 3. Neonates are prone to developing physiological jaundice due to several reasons [3]. At birth, hemoglobin concentrations are higher compared to adult levels, while the life span of circulating RBCs is shorter. Moreover, the hepatic systems of uptake, transport, and conjugation in bilirubin are still immature in the newborn. Increased neonatal gut beta-glucuronidase levels result in enhanced enterohepatic circulation, which further contributes to elevated bilirubin levels. All these factors are pronounced in preterm neonates, while concurrent predisposing conditions may also be present [4,5]. Thus, preterm neonates have a higher incidence of jaundice, increased peak bilirubin levels, and a longer duration of hyperbilirubinemia compared to term neonates.

A disequilibrium between the production, conjugation, and elimination of bilirubin may lead to exacerbation of the normal physiologic transient hyperbilirubinemia. In pathologic jaundice, an underlying medical condition or risk factor leads to hyperbilirubinemia [6]. In this case, jaundice may develop during the first 24 h of life, while bilirubin levels continue to rise rapidly and typically reach higher levels.

Pathologic hyperbilirubinemia should be detected early and treated appropriately, as it bears potentially severe consequences for the neonates. Increased circulating levels of unconjugated bilirubin can be neurotoxic [7]. Bilirubin crosses the blood–brain barrier and may accumulate in the brainstem, hippocampus, cerebellum, globus pallidus, and subthalamic nuclei, causing kernicterus. The broad spectrum of bilirubin-induced neurologic dysfunction (BINT) includes acute and chronic neurologic sequelae. The concentration of unconjugated bilirubin and the duration of hyperbilirubinemia are important determinants of bilirubin neurotoxicity [8]. Preterm or sick neonates and those with a hemolytic condition are at increased risk of bilirubin neurotoxicity [9]. Symptoms of BINT may be subtle, and a high index of suspicion is necessary, in particular for the management of preterm neonates.

Hemolysis, due to both immune and non-immune factors, leads to increased bilirubin production and is the most common cause of pathologic unconjugated hyperbilirubinemia [3]. After the first days of life, bilirubin conjugation and elimination mechanisms have adequately matured, and anemia usually becomes the most significant clinical manifestation of hemolytic conditions [10]. Immune-mediated hemolysis occurs in cases of ABO, Rh, or other RBC antigen incompatibility. The main non-immune causes of hemolytic jaundice include deficiencies of RBC enzymes, defects of the RBC membrane, hemoglobinopathies, and sepsis (Table 1).

This review outlines the most frequent neonatal hemolytic conditions, their specific characteristics, diagnosis, and management options.

## 2. Immune Causes of Unconjugated Hyperbilirubinemia

### 2.1. ABO Blood Group Incompatibility

The ABO system comprises blood types A, B, AB, and O. Type O individuals may produce anti-A or anti-B antibodies when exposed to foreign antigens in foods and bacteria [11]. ABO incompatibility between the mother and the fetus occurs in 15% to 25% of pregnancies. However, symptomatic ABO hemolytic disease occurs in only 1% to 4% of all newborn infants [12]. The incidence is higher among Black populations due to a higher prevalence of anti-A and anti-B antibodies compared to that in Caucasians [13]. At present, ABO incompatibility is the leading cause of neonatal jaundice due to maternal–neonate blood incompatibility [14].

The hemolytic process begins in utero due to transplacental transport of maternal anti-A or anti-B IgG immunoglobulins into the fetal circulation. As a result, fetal RBCs are destroyed by macrophages in the spleen, leading to fetal anemia and hyperbilirubinemia [13]. However, typically, ABO incompatibility becomes concerning after birth. ABO-induced hemolytic disease can occur even during the first pregnancy. Affected neonates mainly belong to blood groups A or B and are born to type O mothers, as naturally occurring anti-A and anti-B antibodies are of the IgG isotype and can cross the placenta. On the contrary, anti-A and anti-B antibodies detected in the serum of group B and A mothers, respectively, are of the IgM isotype [14]. Thus, there are only a few cases of hemolytic disease of the newborn related to ABO incompatibility with a non-O-blood-group mother.

Symptomatic clinical disease is typically characterized by compensated mild hemolytic anemia with reticulocytosis, microspherocytosis, and early-onset unconjugated hyperbilirubinemia [15]. In severe cases, hydrops fetalis may develop. The severity of the hemolysis depends on the quantity and subclass of the maternal IgG isoantibodies [16]. Since A and B antigens are widely expressed in a large variety of tissues, only a small portion of antibodies transferred via the placenta bind to fetal RBCs and cause hemolysis. Additionally, anti-A and anti-B antibodies are usually of the IgG2 subtype, with a lower hemolytic potency [17]. Moreover, ABO blood group antibodies in RBCs are expressed less in the fetus and neonate compared to adults. Consequently, ABO incompatibility generally causes a less severe hemolytic disease compared with Rhesus (Rh) incompatibility.

### 2.2. Rh Incompatibility

The Rh blood group system consists of multiple antigens (over 50), but D, C, c, E, and e are the most commonly identified [18]. Individuals are classified as Rh-positive if they express the D antigen in their RBCs and Rh-negative if they do not. Rh incompatibility is the most well-described cause of hemolytic disease of the fetus and newborn. It begins in utero when a Rh-negative pregnant woman is exposed to a Rh-positive fetus. Initially, IgM antibodies are produced, which do not cross the placenta. The sensitization of the mother depends on multiple factors, including the volume of transplacental hemorrhage, the extent of the maternal immune response, and the concurrent presence of ABO incompatibility [19]. During subsequent exposures, maternal IgG-Rh antibodies are produced and are actively transferred through the placenta, leading to hemolytic disease of the fetus and newborn (HDFN). The risk for symptomatic disease increases with subsequent pregnancies of Rh-positive fetuses, a phenomenon that is not observed with ABO incompatibility [5]. Transplacental hemorrhage and fetomaternal hemorrhage are associated with greater risk of sensitization, while the coexistence of ABO incompatibility reduces the risk of maternal Rh sensitization [19].

The prevalence of Rh-negative-type individuals and the incidence of disease vary around the globe. In North America and Europe, the prevalence of Rh negativity is 15%. It is estimated at 8% in African American women, 4% in African women, and <1% in Asian women [20]. D antigens are highly immunogenic, and as a result, hemolytic disease secondary to Rh incompatibility can be severe. Fetal anemia may lead to increased erythropoiesis and cardiac output and can cause hydrops fetalis and fetal demise, if left untreated [21]. Antenatal screening and prophylaxis with anti-Rh (anti-D) γ globulin has led to a significant decline in reported cases of Rh-hemolytic disease [22]. With these interventions, the mean live birth prevalence of Rh-induced HDFN has declined from 99 per 100,000 to 44 per 100,000 neonates in the United States. Lower rates compared to previous reports were also noted in other countries. However, there still is a 0.1% to 0.4% risk of sensitization in Rh-negative pregnant women, possibly due to antigens other than D. Among newborns with Rh disease and extreme hyperbilirubinemia, the risk of death and stillbirths are 24% and 11%, respectively. Moreover, 13% of affected neonates develop kernicterus [23].

### 2.3. Other RBC Antigen Incompatibility

In addition to ABO and Rh isoimmunization, other subgroup incompatibilities have also been identified among the causes of hemolytic disease and indirect hyperbilirubinemia in neonates [24,25]. The prevalence of RBC antibodies against minor blood group antigens other than anti-D is about 1 in 500 pregnancies [26]. Usually, minor blood group antigens that cause blood incompatibility between the mother and baby include C, c, E, e, Kell, Duffy, Diego, Kidd, Lutheran, Lewis, and MNS antigens [27]. It has been estimated that minor blood group incompatibility accounts for 3–5% of neonatal hemolytic jaundice [28]. Following the widespread use of anti-D γ globulin, minor blood group incompatibility has increasingly contributed to HDFN [26]. In minor blood group incompatibility, the pathophysiological mechanism of isoimmunization is similar to that of Rh incompatibility [28]. However, only one third of cases with minor blood group incompatibility develop a positive direct Coombs’ test, probably due to the weak antigenic properties of minor RBC antigens.

Among all erythrocyte antigens, Kell has been reported as the next most potent immunogenic antigen following anti-D [29]. Notably, in contrast with anemia due to other antibodies, anemia in Kell-HDFN is caused not only by hemolysis but also by the suppression of erythropoiesis. Anti-C has been described as another cause of severe HDFN [26].

Clinical presentation of hemolysis due to minor blood antigens ranges from mild anemia, with reticulocytosis and neonatal hyperbilirubinemia, to marked fetal anemia and hydropic changes [25]. It has been reported that neonates with minor blood group incompatibility have higher levels of total serum bilirubin and more frequently need exchange transfusion compared to neonates from Rh, Rh and ABO, and ABO incompatibility [30]. Advancements in investigation modalities have led to the diagnosis of more cases of minor blood group incompatibility. However, it remains an under-diagnosed entity that should be kept in the differential diagnosis of hemolytic disease in neonates.

#### Hemolytic Disease of the Fetus and Newborn (HDFN)

Maternal–fetal blood group incompatibilities (ABO, Rhesus, and other minor antigens) may result in HDFN, a serious and potentially life-threatening condition [20]. Following alloimmunization, maternal antibodies are transferred in the fetal circulation, potentially triggering hemolysis and subsequent complications. Alloimmunization occurs in cases of fetomaternal hemorrhage during pregnancy, delivery, obstetric emergencies, or procedures [15]. Even an amount of 0.1 mL of fetal blood in the maternal circulation can cause alloimmunization [31].

Antenatally, affected fetuses may present with anemia, polyhydramnios, hepatosplenomegaly, and hydrops fetalis. Hydrops fetalis is usually identified on antenatal ultrasound imaging and is defined as abnormal fluid collection in two or more cavities, including ascites, pleural effusions, pericardial effusion, and generalized skin edema [32]. Hydrops fetalis, with pericardial/pleural effusions and ascites, has a mortality rate of >50% [33]. Mild pleural effusions may cause respiratory distress, while severe effusions can result in lung hypoplasia with poor prognosis for the neonate [34]. Other postnatal manifestations include early-onset unconjugated hyperbilirubinemia, with an increased risk of kernicterus, and mild to severe hemolytic anemia. Severe untreated fetal anemia may lead to cardiovascular shock after birth [35].

For the diagnosis of HDFN, maternal and neonatal blood type (ABO, Rh) and antibody screening is required [36]. A positive Coombs’ test confirms the presence of antibodies in maternal serum (indirect antibody test) or on neonatal RBCs (direct antibody test). However, neonates with a positive Coombs’ test do not always develop severe hyperbilirubinemia, while in some cases of ABO incompatibility and HDFN, direct antibody test is negative [20]. Anemia, reticulocytosis, and signs of hemolysis are often observed in patients with HDFN.

## 3. Non-Immune Causes of Unconjugated Hyperbilirubinemia

### 3.1. RBC Enzymopathies

#### 3.1.1. Glucose-6-Phosphate Dehydrogenase (G6PD) Deficiency

The prevalence of G6PD deficiency is estimated to be approximately 5% on a global scale, affecting over 500,000,000 individuals [37]. Several countries with a high prevalence of G6PD deficiency include this disorder in their neonatal screening programs [38]. The enzyme G6PD catalyzes the reduction of nicotinamide adenine dinucleotide phosphate (NADP) to nicotinamide adenine dinucleotide phosphate hydrogenase (NADPH) during the first step of the pentose phosphate pathway [39]. This compound is essential for the generation of reducing analogues for the most important RBC antioxidant systems [38]. RBCs are exposed to oxidative stress from both endogenous and exogenous sources. In cases of G6PD deficiency, NADPH insufficiency renders RBCs susceptible to oxidative damage and subsequent hemolysis.

The gene coding for G6PD enzyme is located in chromosome X and is inherited through an X-linked recessive pattern [38]. Thus, males are more often affected than females. Clinical expression in heterozygous females is highly variable, depending on G6PD levels determined by the phenomenon of X-chromosome inactivation [37]. The G6PD genetic polymorphism has been extensively studied [40]. By 2020, 230 distinct variants were identified and recorded. Recently, G6PD variants were categorized by the World Health Organization (WHO) into four classes, depending on the degree of enzyme activity and the severity of hemolysis [41]. Class A includes mutations resulting in <20% of normal G6PD activity and chronic non-spherocytic hemolytic anemia. Moderate and severe enzyme deficiencies (<45% of normal activity) presenting with neonatal jaundice and acute hemolytic anemia triggered by certain medications, fava beans, or infection are listed in class B. Class C includes variants with mild or no G6PD deficiency (>60% of normal activity) and no hemolysis. Variants for which there is currently insufficient information regarding clinical manifestations are grouped in Class U. However, enzymatic activity seems to be a poor predictor of clinical presentation, which is rather dependent on genotype [42].

G6PD deficiency is often asymptomatic in most individuals. Nevertheless, its clinical manifestation includes neonatal jaundice; acute hemolytic anemia triggered by infections, medicines, or other agents; and chronic non-spherocytic hemolytic anemia [37].

Neonatal hyperbilirubinemia in the setting of G6PD deficiency is well established and may lead to kernicterus and permanent neurologic damage [38]. More than 20% of the neonatal kernicterus cases in the United States are attributed to G6PD deficiency [43]. It is noteworthy that in most cases, there is little or no evidence of hemolysis [44]. The underlying mechanisms of physiologic neonatal hyperbilirubinemia seem to be aggravated and intensified in G6PD-deficient neonates. Jaundice is rarely present at birth and usually peaks between the second and third days of life [38]. Specific G6PD genetic variants have been reported to be associated with increased risk of neonatal jaundice.

In most G6PD variants, hemolysis usually occurs only after exposure to agents that cause RBC oxidative damage [45]. In the neonatal period, such triggers of acute hemolytic anemia are rarely encountered [38]. However, there are reports of hydrops fetalis and hemolytic anemia in fetuses and neonates of mothers who ingested fava beans prenatally or during breastfeeding [46,47]. Acute hemolysis in the neonatal period may also occur after exposure to environmental oxidative agents [38]. Triggers of acute hemolytic anemia include medications such as anti-malarials, nitrofurantoin, sulfones, and sulfonamides, while rarely, bacterial or viral infections can be implicated.

A small proportion of G6PD-deficient individuals have chronic hemolytic anemia, similar to that of hereditary spherocytosis [48]. They suffer from mild to moderate anemia throughout childhood and adult life. A history of neonatal jaundice, often severe, is present in most cases [37].

The diagnosis of G6PD deficiency should be considered in neonates with excessive hyperbilirubinemia, especially in the absence of other risk factors and even if neither anemia nor hemolysis are present [38,48]. The most commonly used screening test for G6PD deficiency is the fluorescent spot test, a semi-quantitative, easy, and inexpensive method. The spectrophotometric quantitative assay for the measurement of G6PD activity has higher accuracy and sensitivity. In cases where the test is conducted during the acute hemolytic phase, false-negative results may occur due to the high rate of reticulocytes that have an increased level of G6PD activity. Molecular techniques remain the gold standard for diagnosing G6PD deficiency.

#### 3.1.2. Pyruvate Kinase (PK) Deficiency

PK deficiency is an autosomal recessive disorder, with an estimated prevalence ranging from 1 in 120,000 to 1 in 300,000 in the Caucasian population [49]. It is the most common of the glycolytic enzyme deficiencies and the second most common RBC enzymopathy. The PKLR gene, located on chromosome 1q21, catalyzes the conversion of phosphoenolpyruvate to pyruvate in the glycolytic pathway [48]. Therefore, individuals with PK deficiency may have reduced ability to generate adenosine triphosphate (ATP), predisposing them to premature RBC destruction.

The clinical presentation of PK deficiency widely varies, and anemia could be mild or chronic, necessitating transfusions [50]. In the neonatal period, manifestations of PK deficiency range from asymptomatic to a life-threatening condition [51]. Prenatal complications include anemia in utero, hydrops fetalis, intrauterine growth restriction, fetal distress, and preterm birth [52]. The great majority (almost 90%) of neonates with PK deficiency develop severe indirect hyperbilirubinemia and hemolysis, requiring phototherapy and, in many cases, exchange transfusion. Hepatic dysfunction with evolution to liver failure has been reported in neonates [53]. Other more atypical presentations include hyperferritinemia, hypoglycemia, hepatosplenomegaly, respiratory distress, and skin extramedullary hematopoiesis [54]. Older children usually present anemia that may require regular transfusions, as well as gallstones and iron overload [51].

PK deficiency should be considered in neonates with jaundice and signs of non-immune hemolysis [51]. The peripheral blood film examination may reveal echinocytes. Specific diagnostic evaluation should include erythrocyte PK enzymatic activity and genetic testing.

#### 3.1.3. Other RBC Enzymopathies

A total of at least 16 genetically determined conditions are categorized under RBC enzymopathies, most of them being extremely rare [45]. They typically present as chronic hemolytic anemias, with an onset often during the neonatal period. Gene panel testing can contribute to the diagnosis of these still underrecognized conditions.

### 3.2. RBC Membrane Defects

#### 3.2.1. Hereditary Spherocytosis (HS)

Hereditary spherocytosis is caused by defects in the structural proteins of erythrocytes that lead to abnormalities of the cell membrane surface [55]. The resulting deformed RBCs are dysfunctional, with a reduced lifespan. The proteins usually involved are ankyrin-1, α-spectrin, β-spectrin, band 3, and protein 4.2 [56]. Hereditary spherocytosis is the most common type of hereditary hemolytic condition in individuals of Northern European decent. Its prevalence is estimated to range from 1/1000 to 1/5000 births. Approximately 75% of cases are associated with an autosomal dominant inheritance pattern, while the remaining 25% consist of recessive forms and de novo mutations. Mutations in genes encoding ankyrin, β-spectrin, and band 3 often result in autosomal dominant HS [57].

The clinical spectrum of HS widely varies from asymptomatic individuals to severe hemolysis and life-threatening, transfusion-dependent anemia [58]. In the prenatal period, affected fetuses may present with severe anemia and hydrops fetalis [59]. Hyperbilirubinemia, often prominent, is the most common problem in neonates with HS. The triad of anemia, splenomegaly, and jaundice, typical in older children and adults with HS, is rare in neonates [58]. More than 50% of neonates with HS are not anemic during the first week of life.

The genetic and phenotyping heterogeneity of HS make its diagnosis during the first year of life difficult. A family history of HS should be sought and documented in neonates with non-immune hyperbilirubinemia [58]. Reticulocytosis and indexes of hemolysis may not be remarkable in neonates, and spherocytes are less frequently observed in them compared to in older children [55]. A complete blood count with RBC indices typically reveals an elevated mean corpuscular hemoglobin concentration (MCHC) and a low mean corpuscular volume (MCV), while the peripheral blood smear may highlight the presence of spherocytes and polychromasia. The “Neonatal HS Index” is calculated by dividing MCHC by MCV, and a value > 0.36 is highly indicative of HS [59]. The traditional diagnostic tool for HS, osmotic fragility, is less reliable in neonates [60]. Osmotic gradient ektacytometry measures erythrocyte deformability but is only available in specialized laboratories. Eosin-5’ maleimide, a flow cytometry-based test, identifies membrane protein deficiencies, and has been used for the diagnosis of RBC membrane defects. Incubated osmotic fragility and flow cytometric methods for the assessment of osmotic fragility may be helpful [55]. Genetic testing is confirmatory of the diagnosis in selected cases and useful for family counseling.

#### 3.2.2. Hereditary Elliptocytosis and Hereditary Pyropoikilocytosis

Hereditary elliptocytosis and pyropoikilocytosis are heterogeneous disorders caused by mutations in genes encoding α-spectrin, β-spectrin, and protein 4.1R [61]. Hereditary elliptocytosis, an autosomal dominant condition, has a worldwide distribution. Its estimated prevalence is 3–5/10,000, with a higher incidence in malaria-endemic regions. Elliptically shaped RBCs have reduced membrane stability and life span. Hereditary elliptocytosis is often asymptomatic; however, some patients present with hemolysis, anemia, hyperbilirubinemia, and splenomegaly. In neonates, hereditary elliptocytosis may manifest as hydrops fetalis, jaundice, and severe hemolytic anemia.

Hereditary pyropoikilocytosis, a rare, autosomal recessive condition, is characterized by pyknocytes, elliptocytes, and fragmented RBCs on the peripheral smear [62]. Affected neonates usually develop severe hemolytic anemia, requiring transfusions during the first months of life [63].

Diagnosis of these conditions in the neonatal period is based on family history, complete blood count, morphology of erythrocytes, and indices of hemolysis [61]. Sodium dodecyl sulfate-polyacrylamide gel electrophoresis and ektacytometry may be helpful, while genetic analysis is invaluable in cases with ambiguous clinical presentation.

#### 3.2.3. Rarer RBC Membrane Disorders

Hereditary xerocytosis or dehydrated hereditary stomatocytosis, overhydrated hereditary stomatocytosis, and South East Asian ovalocytosis are rarer disorders of the erythrocyte membrane [62]. In general, they have similar clinical presentations and are diagnosed with RBC morphology, ektacytometry, and next-generation sequencing.

### 3.3. Hemoglobinopathies

Hemoglobinopathies are caused by mutations in genes encoding hemoglobin chain proteins that result in quantitative or qualitative defects [64]. Hemoglobinopathies are generally autosomal recessive disorders, characterized by ineffective erythropoiesis and hemolytic anemia. Although most of these conditions manifest later, some of them present during fetal and neonatal life. Due to large amounts of fetal hemoglobin, sickle cell disease (structural β globin chain abnormality), β-thalassemia (impaired β-chain production), and other β globin chain disorders typically become clinically evident in older infants.

A-thalassemia (impaired α chain production) is the main entity associated with neonatal jaundice. It results from mutations in one or more of the four α globin genes. If one or two genes are mutated, affected individuals are asymptomatic carriers or mildly anemic. Hemoglobin H disease is the case of mutations in three genes, and α globin chains are significantly decreased [65]. Therefore, the excess γ and β chains form unstable tetramers that lead to hemoglobin precipitation in RBCs and hemolysis. If four genes are mutated, the absolute lack of α chains results in hydrops fetalis and death in utero or after birth.

Rarely, mutations in globin chains can lead to unstable hemoglobin variants (e.g., hemoglobin Hasharon or F Poole) [64]. Precipitation in RBCs as Heinz bodies follows, along with intravascular and extravascular hemolysis. Clinical presentation varies, but unstable hemoglobinopathies often manifest as neonatal hemolytic anemia and hyperbilirubinemia.

Hemoglobinopathies are included in some neonatal screening programs [64]. Classical electrophoresis, or most recently, newer technologies including high-performance liquid chromatography, capillary electrophoresis, and isoelectric focusing, are used for the diagnosis of hemoglobinopathies in neonates [66]. Molecular genetic testing can confirm the diagnosis [64].

### 3.4. Congenital Dyserythropoietic Anemia

Congenital dyserythropoietic anemias are rare inherited disorders of erythropoiesis [67]. Dyserythropoiesis results in the premature destruction of RBCs and hemolytic anemia [68]. Congenital dyserythropoietic anemia types I and IV may cause fetal and neonatal anemia and neonatal jaundice [67].

### 3.5. Sepsis

Neonates with sepsis are predisposed to severe jaundice due to both excess RBC destruction and hepatocellular dysfunction [69]. Infections may lead to severe jaundice or kernicterus in 2% of neonates in Europe and North America, 14% in Africa, and 31% in Asia. It has been reported that neonates with sepsis are at increased risk for severe hyperbilirubinemia in low-income and middle-income countries compared to high-income countries. Moreover, sepsis is considered a neurotoxicity risk factor associated with hyperbilirubinemia [9].

Unconjugated hyperbilirubinemia is mainly attributed to sepsis-induced oxidative damage of erythrocytes [69]. Triggers of hemolysis during sepsis include disseminated intravascular coagulation, complement activation, microvascular stasis, invasion of the amphiphilic lipopolysaccharide into the RBC membrane with corresponding changes in membrane properties, hemolytic pathogens and pore-forming toxins, RBC metabolic disorders, and RBC apoptosis [70,71].

### 3.6. Other Causes

Low vitamin E levels have been associated with increased bilirubin in neonates, and supplementation with vitamin E has been studied for the treatment of unconjugated hyperbilirubinemia [72]. Vitamin E is part of many cellular antioxidant systems, and it seems to slow the hemolytic process in RBCs.

Infantile pyknocytosis is a rare, non-immune, hemolytic condition of unknown and probably multifactorial etiology [73]. It should be suspected in neonates with hemolytic jaundice or anemia, a significant rate of pyknocytes in the peripheral blood smear, and evidence of Heinz bodies. Its diagnosis is based on the exclusion of other possible causes, and its resolution is spontaneous.

## 4. Diagnostic Approach

Although neonatal jaundice is commonly observed, its cause very frequently remains unidentified, while many cases of severe indirect hyperbilirubinemia are classified as idiopathic [10]. Even in cases of kernicterus, the cause of jaundice is not determined in over half of the patients [74]. A substantial number of these neonates most probably have an underlying hemolytic condition. Early identification of neonatal hyperbilirubinemia causes can prevent short-term and long-term complications and improve outcomes [1].

An initial assessment of the risk of HDFN should be completed antenatally, with ABO blood group and Rh-typing and antibody screenings of all pregnant women during their first prenatal visit [36]. After delivery, the American Academy of Pediatrics recommends that all neonates should be visually assessed for jaundice every 12 h until discharge [9]. For neonates with certain risk factors for hyperbilirubinemia, including lower gestational age and hemolysis from any cause, closer monitoring is required.

A detailed family and pregnancy history, physical examination of the neonate, and further investigations are necessary in the differential diagnosis of neonatal jaundice. Diagnostic evaluation of a neonate with pathological hyperbilirubinemia includes maternal and neonatal AΒO and Rh-status, direct antibody test, complete blood count, reticulocyte count, peripheral blood smear, and G6PD testing [10].

Establishing the presence of hemolysis is a crucial step. Early onset of the jaundice during the first 24 h and rapid bilirubin elevation (>0.3 mg/dL per hour during the first day of life and >0.2 mg/dL per hour thereafter) is indicative of hemolysis. Hemoglobinuria without RBCs in the urine, absence of serum haptoglobin, elevated lactic dehydrogenase (LDH), anemia, increased reticulocyte count, and abnormal RBC morphology in the peripheral blood smear are also suggestive of a hemolytic process [75]. However, each of these tests has its limitations during the first days of life; thus, detection of hemolysis in neonates is not always straightforward.

End-tidal carbon monoxide, corrected for ambient carbon monoxide (ETCOc), has emerged as a useful tool for both the identification of hemolysis and the quantification of its rate [76]. When available, measurement of ETCOc may be superior to other diagnostic methods for neonatal hemolytic jaundice, accurately reflecting the destruction of RBCs and subsequent bilirubin production. ETCOc levels are increased in hemolysis of any cause. A cut-off value of 2.0 ppm is typically used for detecting hemolysis in neonates.

Morphologic abnormalities of RBCs are common in neonates with jaundice and may help identify its cause [77]. Spherocytes are characteristic of either ABO hemolytic disease or HS, while elliptocytes are observed in hereditary elliptocytosis. Bite and blister cells are present in G6PD deficiency and unstable hemoglobinopathies. Echinocytes are usually found in patients with PK deficiency. Schistocytosis may be encountered in hemolysis of any cause but is usually indicative of disseminated intravascular coagulation, large hemangiomas, and other microangiopathic conditions.

Eosin-5’ maleimide flow cytometry may be helpful in the diagnosis of RBC membrane defects, especially HS [10]. Hemoglobin electrophoresis can be used for the detection of hemoglobinopathies [64].

Lately, next-generation sequencing has rendered the diagnosis of previously unidentified neonatal hemolytic conditions feasible [10]. Targeted next-generation sequencing panels detecting underlying genes that result in neonatal hyperbilirubinemia have been developed in clinical reference laboratories [75]. These panels have helped identify cases with erythrocyte membrane disorders or enzyme deficiencies that were previously diagnosed as ‘idiopathic’, allowing for a more focused approach to the condition, needs, and treatment options of the patients and their families. Turnaround time, availability, and cost-effectiveness should also be taken into account when deciding on the diagnostic methodologies to be used in a specific patient.

**Figure 1:**
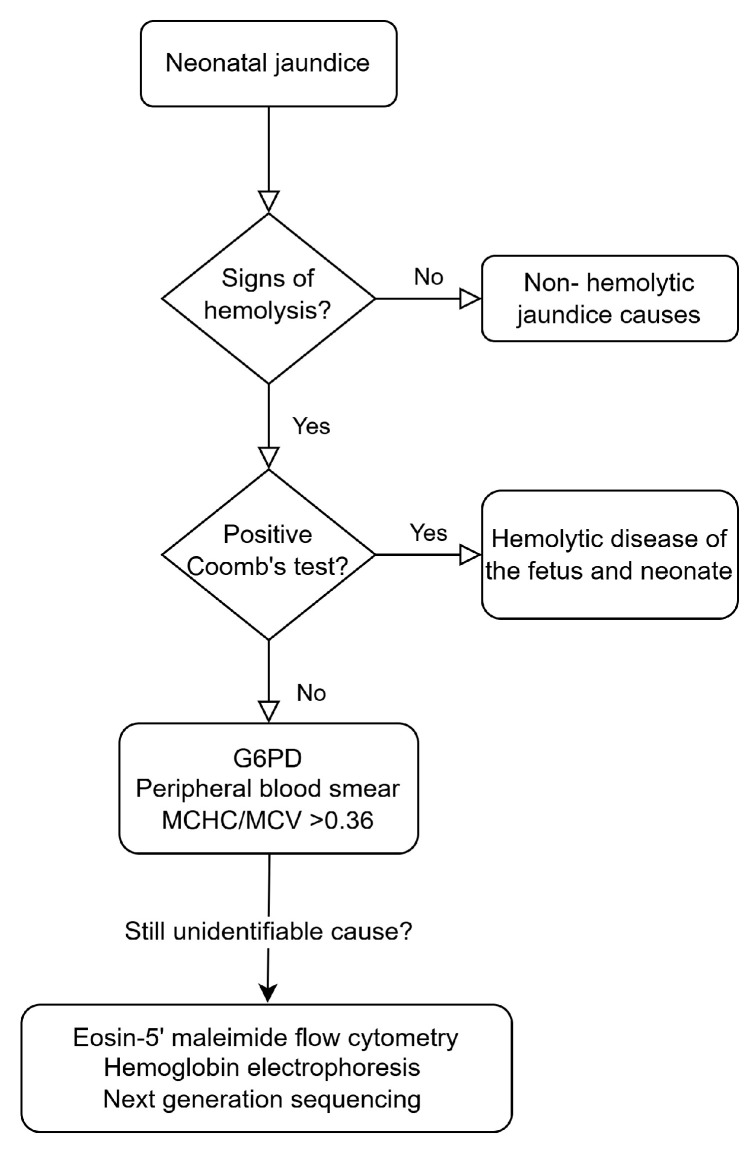
Diagnostic approach to neonatal jaundice.

Figure 1 presents a simplified diagnostic approach to neonatal hemolytic jaundice.

## 5. Management

In cases of ABO or Rh isoimmunization, monitoring for HDFN should begin during pregnancy with Doppler ultrasonography [78]. If fetal anemia is detected, intrauterine transfusion may be required.

Phototherapy is the mainstay of management for neonates with unconjugated jaundice, as highlighted in the recent guidelines of the American Academy of Pediatrics [9]. Through photochemical reactions, bilirubin is transformed to more easily excretable molecules, and its concentration is reduced. The effectiveness of phototherapy depends on its intensity and on the surface area of the body exposed to it. Phototherapy is safe, with only mild side effects [1]. Adequate hydration should be ensured. In cases of hemolytic jaundice, or if bilirubin is rapidly increasing, intensive phototherapy may be used. Treatment threshold graphs have been developed to guide phototherapy, based on gestational age, day of birth, and the presence of neurotoxicity risk factors [9]. Isoimmune hemolytic disease is a recognized bilirubin neurotoxicity risk factor. Although it is unclear whether neonates with other hemolytic conditions are also at increased risk of bilirubin neurotoxicity, they are presumed to be.

Treatment of neonates who fail to respond to intensive phototherapy and their bilirubin levels remain above exchange transfusion thresholds will need to be escalated, as per the updated guidelines of the American Academy of Pediatrics [79]. Graphs are available to define exchange transfusion thresholds dependent on gestational age, postnatal hour, and the existence of bilirubin neurotoxicity factors [9]. In exchange transfusion, aliquots of neonatal blood are replaced with cross-matched donor blood, resulting in removal of bilirubin and hemolytic antibodies from neonatal circulation. This intervention has potentially severe complications, and close monitoring of the neonate is necessary throughout the procedure.

A few adjuvant pharmacological agents have been studied for use in the management of indirect hyperbilirubinemia, along with phototherapy [80]. In neonates with severe hemolytic jaundice due to HDFN, high doses of intravenous immunoglobulin have been associated with conflicting results and safety concerns; therefore, the American Academy of Pediatrics recommends its use when levels of bilirubin reach the escalation-of-care threshold [9]. Phenobarbital has been traditionally used in the treatment of neonatal hyperbilirubinemia as it enhances bilirubin metabolism and urinary excretion [81]. Its slow action time and safety profile limit its use. Albumin binds to free bilirubin, increasing its excretion and reducing its neurotoxicity. Infusion prior to exchange transfusion appears to be safe and effective in decreasing the need for exchange transfusion and shortening the duration of phototherapy [82]. The addition of oral probiotics/prebiotics/synbiotics to phototherapy has been associated with a higher bilirubin reduction rate in a number of studies but cannot be routinely recommended [80]. Metalloporphyrins block the activity of heme oxygenase and have been studied in neonates with hyperbilirubinemia; however, their efficacy and safety are still unclear [81]. Fibrate, a lipid-lowering drug, enhances bilirubin conjugation and excretion [83]. Although some studies have reported favorable results, further research is necessary [80].

## 6. Conclusions

Hemolytic jaundice remains one of the most common conditions encountered during neonatal life. Its timely and appropriate detection, diagnosis, and management are extremely important for the well-being of the child, the whole family, and public health. Monitoring neonates for the presentation of pathologic hyperbilirubinemia and prompt treatment of those at risk of neurotoxicity is a significant step. However, the identification of hemolysis and differential diagnosis of hemolytic causes of jaundice need to be equally kept in focus in everyday clinical practice. Contemporary diagnostic tools are available to help clarify previously ‘idiopathic’ cases.

## Figures and Tables

**Table 1:** Causes of hemolysis in the neonate.

Immune-mediated hemolysis ABO incompatibility Rh incompatibility Other RBC antigen incompatibility
Non-immune hemolytic causes Deficiencies of RBC enzymes (G6PD deficiency, pyruvate kinase deficiency) Defects of RBC membrane (hereditary spherocytosis, elliptocytosis, pyropoikilocytosis, xerocytosis, stomatocytosis) Hemoglobinopathies Congenital Dyserythropoietic Anemia Sepsis Extravasated blood collection Vitamin E deficiency Infantile pyknocytosis
